# Metamaterial foundation for seismic wave attenuation for low and wide frequency band

**DOI:** 10.1038/s41598-023-27678-1

**Published:** 2023-02-09

**Authors:** Arpan Gupta, Rishabh Sharma, Aman Thakur, Preeti Gulia

**Affiliations:** 1 Acoustics and Vibration Laboratory, School of Engineering, Indian Institute of Technology, Mandi, Himachal Pradesh India; 2 Department of Mechanical Engineering, National Institute of Technology, Agartala, Tripura 799046 India

**Keywords:** Environmental sciences, Natural hazards, Engineering, Mathematics and computing, Physics

## Abstract

Metamaterials are periodic structures made by repeating a unit cell. Such a structure shows frequency-specific wave attenuation behaviour. In this work, a 2D metamaterial foundation is proposed for the seismic protection of buildings. The paramount challenge is to offer low frequency attenuation (~ 2–8 Hz), which is the dominant excitation during an earthquake. Based on the parametric study performed, a new type of metamaterial structure was proposed. It was found that the foundation consisting of repeating circular scatterers made of steel and plumbum embedded in rubber matrix can provide low and wide frequency wave attenuation from 2.6 to 7.8 Hz. The computational model of the structure was subjected to transient excitation against three pre-recorded earthquake excitations. The result showed that the novel foundation can resist the propagation of the seismic wave to the structure. Further, the response of a 2D building frame with metamaterial foundation was compared to a concrete foundation exposed to different earthquake excitations. The results are very promising as the frame vibration on the metamaterial foundation was significantly less than the same frame on the concrete foundation. The presented work opens the path to new research and development of seismic metamaterial foundation for earthquake attenuation.

## Introduction

Daily more than ten thousand structures are built across the globe. Fabricated by a plethora of concrete, steel, and other construction material, these rigid appearing structures are susceptible to earthquakes. The vibrational energy produced during earthquakes causes damage to these structures leading to a tremendous loss to humanity. One way to avoid structural damage is by using foundations made up of metamaterials. Metamaterials, as the name suggests, are superlative materials with properties beyond natural materials. Recent studies^1–3^ show that these metamaterials, also referred to as periodic materials, may reduce vibrations in structural elements. These metamaterials can be used to design a building foundation to eliminate the effect of earthquake^4,5^. Kacin et al.^6^ studied a seismic metamaterial with triangular lattice structure using finite element simulation and validated the results with the experiments. The study shows that the presented metamaterial effectively attenuates the surface wave at 8 Hz. Brule et al.^7^ bored the cylindrical inclusion in the soil and tested its effectiveness against surface wave of frequency 50 Hz with lateral amplitude 0.014 m. The periodic constant of bored voids in soil was comparable with the wavelength of the incident wave. It was found that the structured soil with deep bore hole acted as an effective shield for the 50 Hz surface waves. As a novel seismic isolation technique, structural foundations made with periodic materials possess the inherent ability to forbid the seismic wave energy transmission to the superstructure^8–10^. This ability of the periodic foundation to forbid seismic wave energy transmission comes from the fact that energy travels in solid in the form of waves, and periodic materials don't allow waves of a certain frequency to pass through them. Thakur et al.^11^ studied bandgaps of three-dimensional periodic structures. The study showed that the three-dimensional periodic structure having specific attenuation zones reduces the amplitude of waves whose frequency lies in attenuation zones. Nouh et al.^12^ studied a meta-material beam composed of periodically arranged viscoelastic membrane unit cells. The results showed that the structure provides a remarkable wave amplitude attenuation at very low frequency ranges. Specific frequency gaps called bandgaps are generated when waves travel through these periodic materials. If travelling waves have frequency in the bandgap region, then periodic material doesn’t allow that wave to travel through it. Huang et al.^13^ designed a metamaterial for structural vibration and elastic wave absorption. They used mass in mass spring system to make unit cell of the metamaterial. The results showed the band gaps at low frequency with negative effective mass and negative effective density in that particular frequency range. A periodic material is a composite material composed of infinite repetitions of one unit cell, where a unit cell is the most fundamental building block of a periodic material. The unit cell consists of a combination of different materials fabricated to a particular geometry, reflecting the characteristics of its frequency band gap zones. Sharma et al.^14^ designed a beam composed of periodically arranged internal resonators. The result showed two band gaps; one is associated with the Bragg’s band gap, and the second is associated with the resonance frequency of resonators. Periodic foundations which are used to solve real engineering problems are made up of finite repetitions of the unit cell and promise attenuation zone overlapping with the frequency bandgap of a unit cell. Hsu^15^ used a series of stepped resonators on a thin slab to design a phononic crystal and performed a finite element study to compute the transmission spectra of the structure. It was found that the structure exhibited a low frequency forbidden zone, which could be tuned by changing the resonating structure and periodic symmetry of the structure^16^. Jensen^17^ studied the effect of boundary conditions and damping on the vibrational characteristics of a periodic structure. It was shown that a moderate amount of damping does not affect bandgap. In contrast, strong damping led to vanishing the presence of bandgap, and band properties were sensitive to the various boundary conditions. Zhao et al.^18^ designed a double vibrator using pillared periodic structural plate. The result showed that the height of periodic units had a major effect on the position of band gaps. Oudich et al.^19^ performed an experimental study of a 2-D phononic stubbed plate. The periodic unit was composed of thin aluminium plate stubbed with silicone rubber. The proposed structure showed the existence of local resonance gap at low frequencies. Qian et al.^20^ studied a double panel system with periodic arrangement of mass spring resonators. The result showed that adding spring near the resonators leads to widen the bandwidth at lower frequencies.

**Figure 1:**
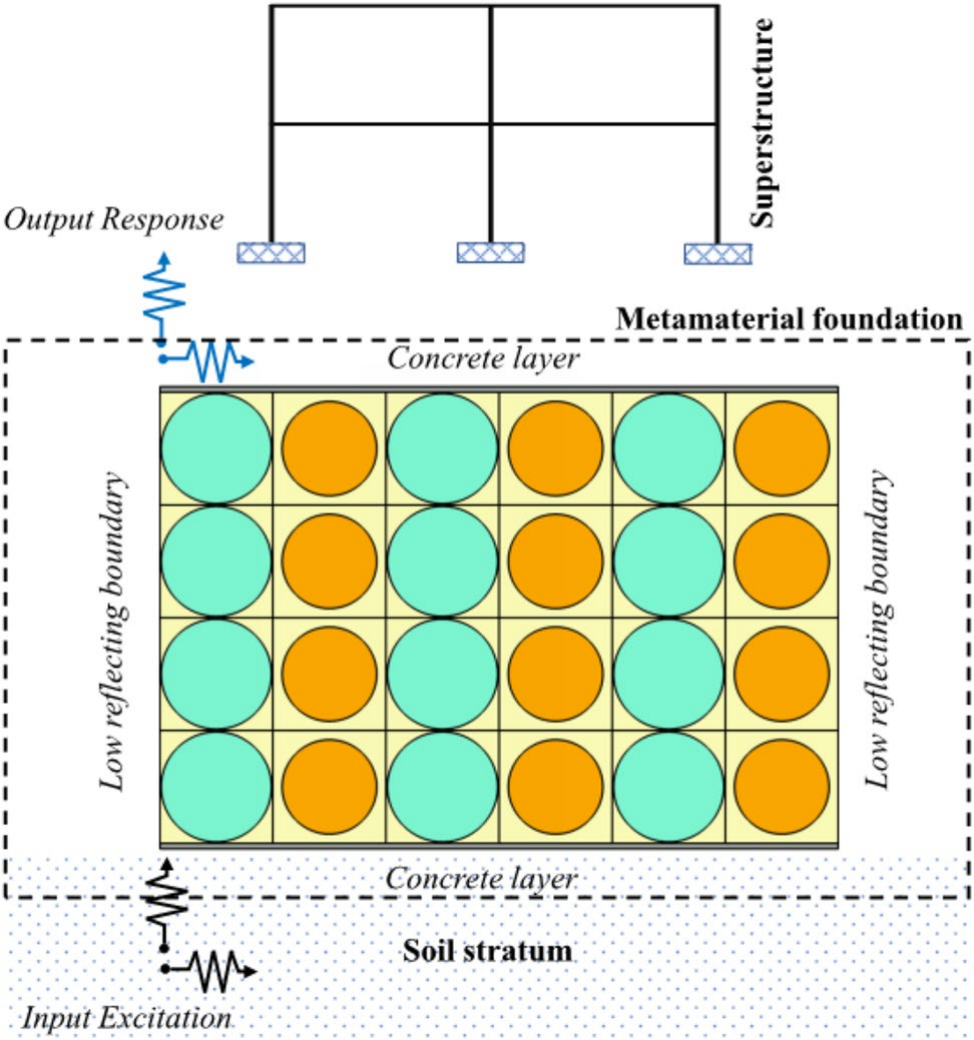
A periodic foundation structural system.

This study aims to design a two-dimensional metamaterial foundation with wider bandgaps in low-frequency regions. A periodic foundation (refers to Fig. 1) made up of two distinct unit cells has been studied and designed. The unit cells are made up of different materials with different geometric properties. Unit cell with circular inner scatterer is a very fundamental design, emulating the periodic arrangement of atoms in solids. The starting point of such structure design is the fundamental frequency that is intended to be attenuated. A simple calculation based on Bragg’s frequency can give an initial estimate of the design. Other designs can be of any other shape of the scatterer in square lattice. The shape of scatter can be square, triangular etc., some of the performance analysis have been reported by Cheng and Shi^21^. Throughout the context of this paper, the terms frequency bandgap, attenuation zones, periodic structures/metamaterial foundations will be commonly used. Significant emphasis on these terminologies with illustrations has been made to get the correlation between them.

## Finite element study

In this study, the periodic material used to make a two-dimensional foundation for wave attenuation comprises two different types of unit cells. Both unit cells are square in shape with a circular inner scatterer. The outer layer of both the unit cells is made of rubber, whereas the inner circular core of one unit cell is steel and the other is plumbum. The engineering properties of the materials of the unit cells are as per Table 1.

**Table 1:** Material properties.

Material	Youngs modulus E (Pa)	Poisson ratio υ	Density ρ (kg/m^3^)
Rubber	1.37 × 10^5^	0.463	1300
Plumbum	1.646 × 10^10^	0.4	11,600
Steel	2.1 × 10^11^	0.3	7850
Concrete	3 × 10^10^	0.2	2500

### Governing equations

Wave propagation in a two-dimensional inhomogeneous solid^22^ when the material used is assumed to be continuous, perfectly elastic, isotropic with small deformations, and has zero damping is governed by Eq. (1).

1 $$ \rho \left( r \right)\frac{{\partial^{2} u_{i} }}{{\partial t^{2} }} = \frac{\partial }{{\partial x_{i} }}\left[ {\lambda \left( r \right)\frac{{\partial u_{j} }}{{\partial x_{i} }}} \right] + \frac{\partial }{{\partial x_{j} }}\left[ {\mu \left( r \right)\left( {\frac{{\partial u_{j} }}{{\partial x_{i} }} + \frac{{\partial u_{i} }}{{\partial x_{j} }}} \right)} \right] $$

In the above equation, $u$ is the displacement vector, $r$ is the coordinate vector, $\rho$ is the density, $t$ is the time parameter, $\lambda$ and $\mu$ are the Lame’s constants, and Eq. (2) gives their representation in terms of young’s modulus $E$ and poison’s ratio $\upsilon$.

2 $$ \lambda = \frac{\upsilon E}{{\left( {1 + \upsilon } \right)\left( {1 - 2\upsilon } \right)}},\mu = \frac{E}{{2\left( {1 + \upsilon } \right)}} $$

### Periodic boundary conditions

Bloch’s theory is used to solve the wave propagation equation given by Eq. (1), and its solution is given by Eq. (3).

3 $$ u\left( {r,t} \right) = e^{{i\left( {K \cdot r - \omega t} \right)}} u_{k} \left( r \right) $$

In the above equation, *K* denotes the wave vector in reciprocal space, *ω* represents angular frequency, and ${u}_{k}\left(r\right)$ indicates the wave amplitude, which is a periodic function^22^ given by Eq. (4).

4 $$ u_{k} \left( r \right) = u_{k} \left( {r + A} \right) $$

**Figure 2:**
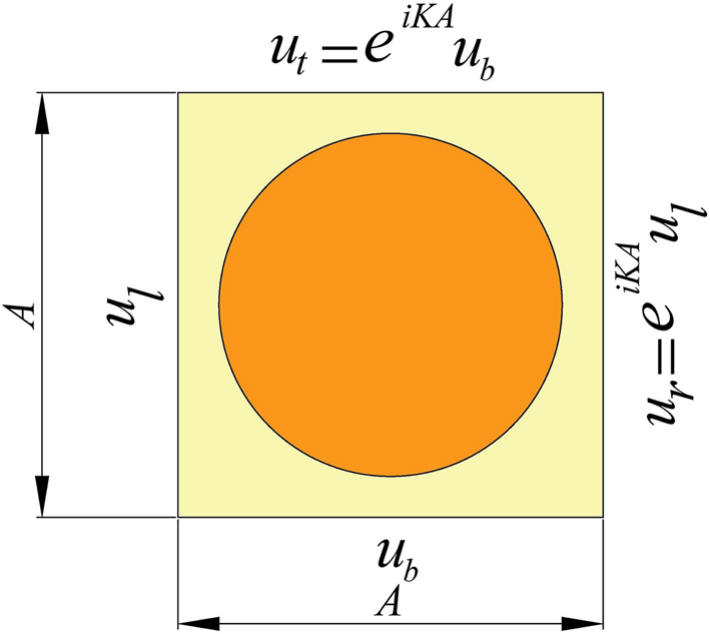
Periodic Boundary conditions of the two-dimensional periodic structure.

In the above equation, *A* is the periodic lattice constant or the distance between two scatterers, shown in Fig. 2.

Due to periodicity, dispersion relations of the infinite periodic structure, which leads to bandgaps, can be obtained using one unit cell with periodic boundary conditions. By substituting Eq. (4) into Eq. (3), periodic boundary conditions can be obtained and are given by Eq. (5) and are shown in Fig. 2^23^.

5 $$ u\left( {r + A,t} \right) = e^{iKa} u\left( {r,t} \right) $$

### Dispersion relationship

The wave equation is converted into an eigenvalue equation^21^ given by Eq. (6), also known as the dispersion relation using periodic boundary conditions as depicted in Fig. 2.

6 $$ \left( {\Omega \left( K \right) - \omega^{2} M} \right)U = 0 $$

**Figure 3:**
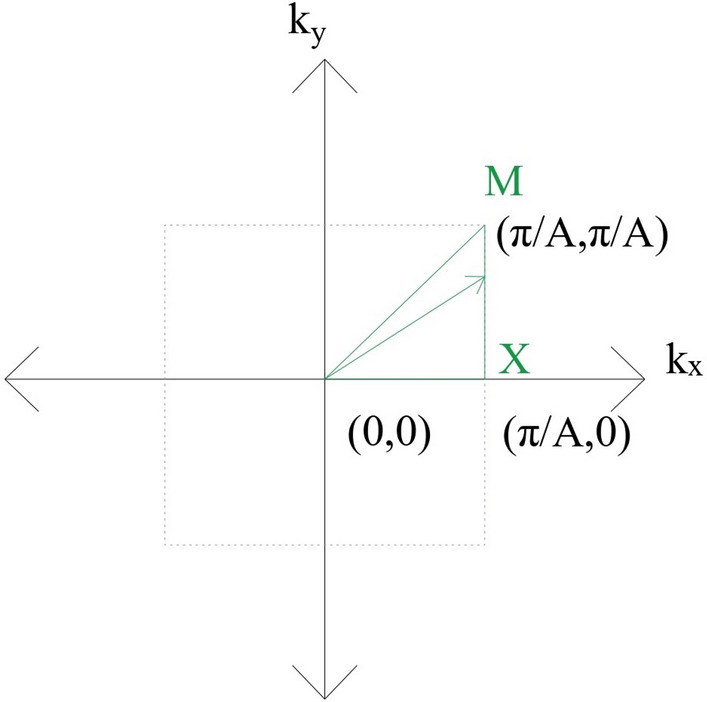
First irreducible Brillouin zone.

In the above equation, $\Omega$ is the stiffness matrix, *M* is the mass matrix, and *U* is the displacement matrix of the unit cell. To get the dispersion relation, Eq. (6) is solved for every value of the *K* vector, which is varied along the boundary of the first irreducible Brillouin zone, which is enclosed by a triangle *ΓXMΓ* as shown in Fig. 3.

## Bandgap of an infinite periodic structure

Dispersion relation graphs help to find bandgaps of infinite periodic structures. The vertical axis of the dispersion relation graph has frequency values and has two frequency regions; one is called pass band, and the other is stop band. In the pass band region, eigen frequencies correspond to each wave vector, whereas, in the stop band region, no eigen frequency is present corresponding to any wave vector. Waves whose frequencies lie in pass band region can pass through the periodic structure, whereas waves whose frequencies lie in the stop band region cannot pass through the periodic structure. In this study, FEA analysis software, COMSOL Multiphysics 5.2, is used to solve dispersion equations and obtain dispersion relation graphs. The unit cell is modelled, and suitable material properties are assigned to it. Periodic boundary conditions are applied on the opposite edges of the unit cell and Floquet periodicity is chosen as type of periodicity. Further, the parametric sweep is used to vary the value of the wave vector in eigen frequency analysis.

**Figure 4:**
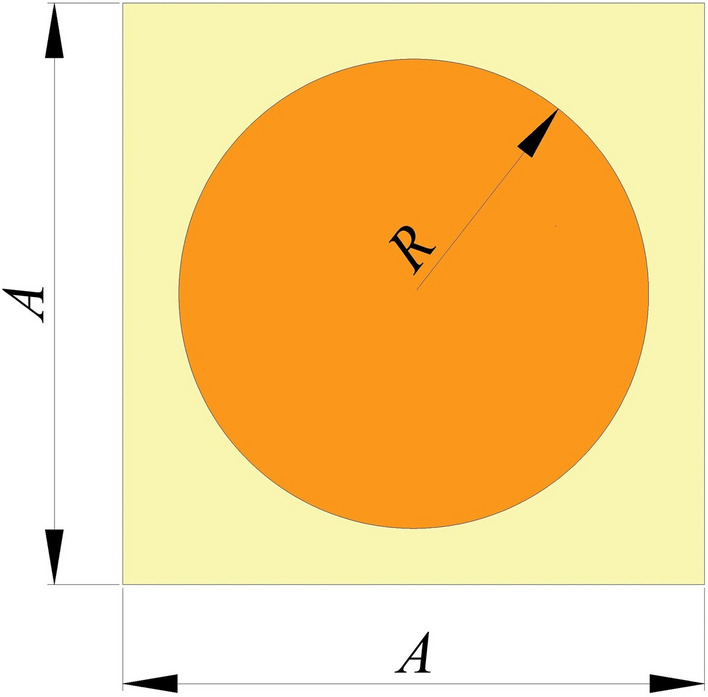
Unit cell with circular inner scatter.

To assure the correctness of the adopted method of finding band gaps, the band gap of the unit cell shown in Fig. 4 is calculated and compared with the literature.

**Figure 5:**
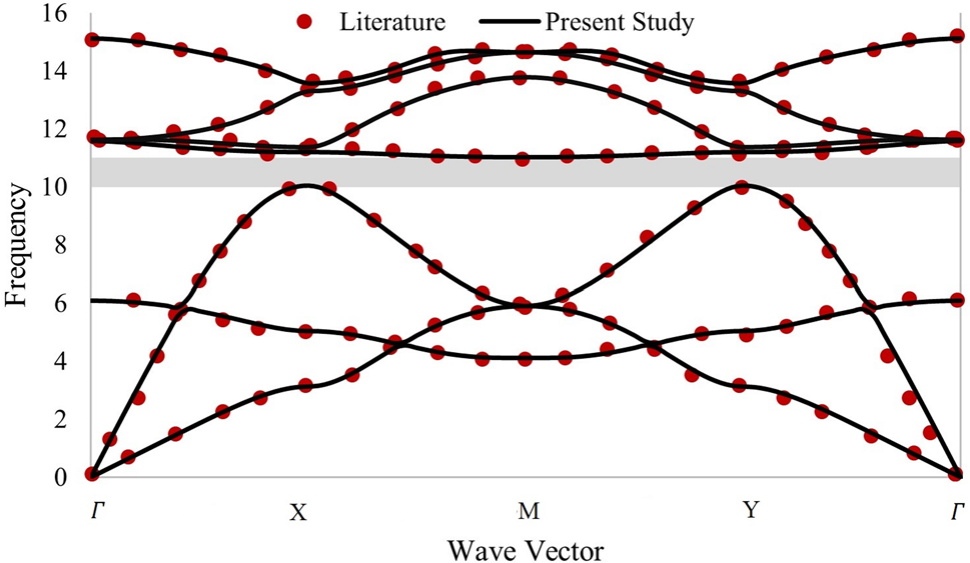
Dispersion relation of rubber concrete unit cell.

For the unit cell, as shown in Fig. 4, the value of *A* is 1 m and *R* is 0.4 m. The outer matrix of the unit cell is made up of rubber, whereas the inner scatter is made of concrete, whose properties are as per Table 1. Figure 5 shows that the obtained result has a good agreement with the literature^21^.

### Parametric study

While deciding the dimensions, geometries, and arrays, various factors have to be taken into account. In periodic structures, the lattice constant can be associated with the Bragg’s frequency or the resonant frequency. Before deciding the lattice constant, one should be clear about which frequencies should be attenuated. The lattice constant should be comparable with the wavelength of the wave that is to be attenuated.

Increasing the number of unit cells in the array causes an increase in the amplitude of attenuation. Based on the highest amplitude of the wave, an array can be determined. In this instance, a parametric analysis was also done to determine the dimensions and array of the metamaterial.

A parametric study is done to identify the unit cell's geometry influence on the band gaps.

For the parametric study, packing ratio *p* is introduced, whose value is given by Eq. (7).

7 $$ p = \frac{{\pi R^{2} }}{{A^{2} }} $$

**Figure 6:**
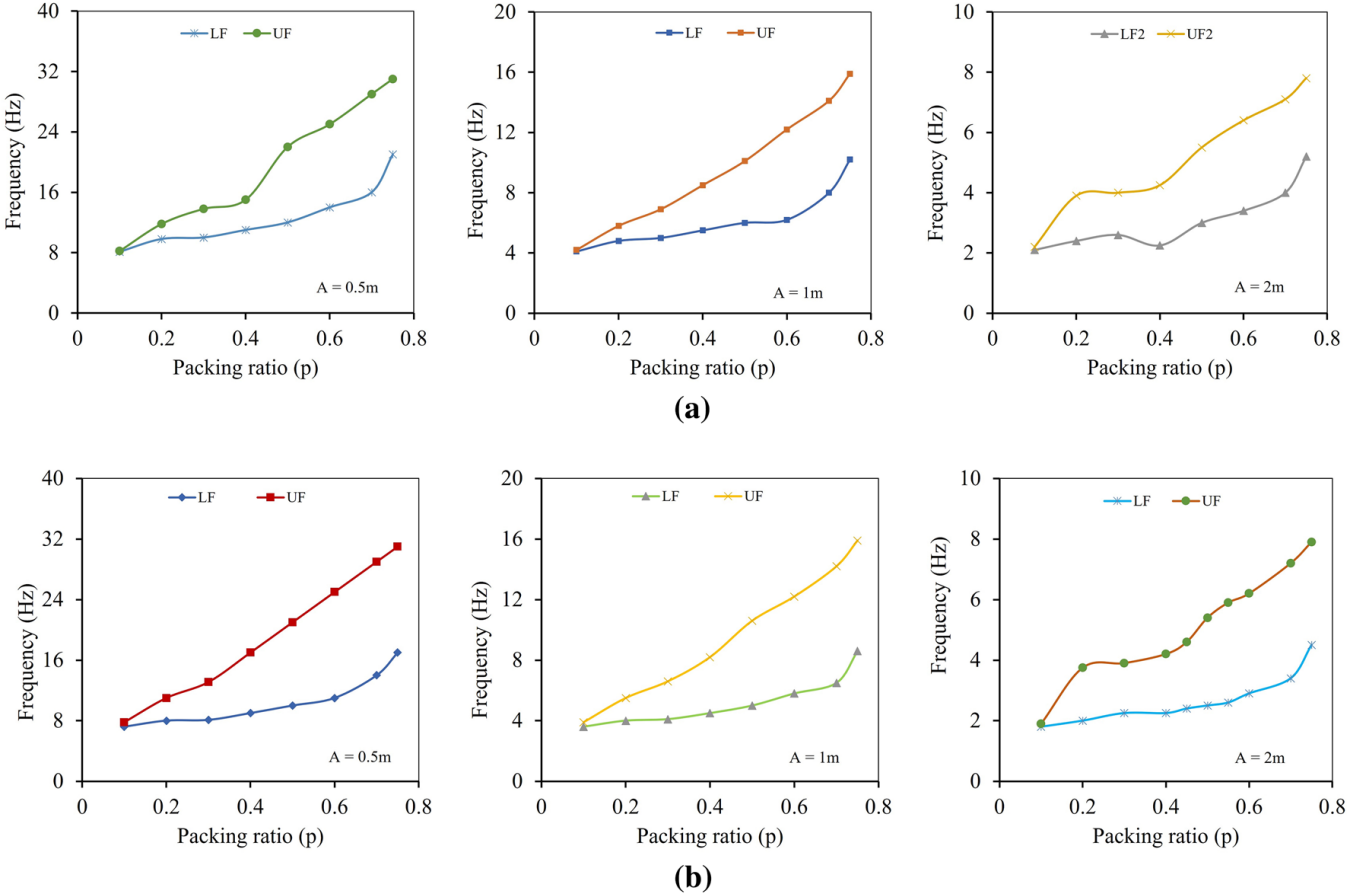
Effect of packing ratio on bound frequencies of attenuation zones of (**a**) Rubber-Steel unit cell (**b**) Rubber-Plumbum Unit cell (UF and LF represent the upper and lower frequencies of stop band).

Keeping the value of *A* constant, radius of the inner circular scatterer of both the unit cells is varied to increase or decrease the value of the packing ratio. Figure 6 represents the variation of frequencies of band gaps of both unit cells with increasing value of packing ratio for three different values of *A*.

**Figure 7:**
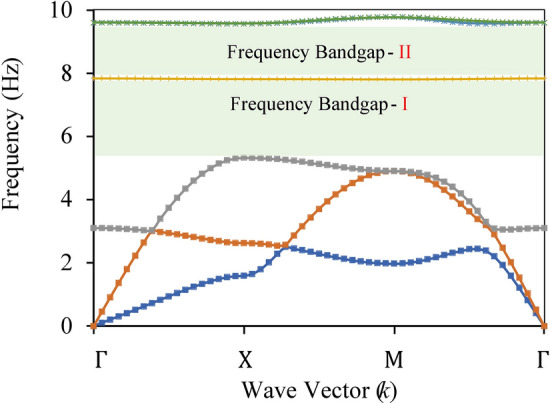
Dispersion relation of rubber steel unit cell.

It is evident from the above figure that for both the unit cells, the bound frequencies of the attenuation zone decrease as we increase the size of the unit cell. So, for this study, we have adopted a unit cell of length 2 m to make the periodic foundation. For the 2 m square unit cell made up of rubber and steel, at *p* = 0.75 for frequency band gap 1, the lower bound frequency of the attenuation zone is 5.2 Hz, and the upper bound frequency is 7.8 Hz, as shown in Fig. 7.

**Figure 8:**
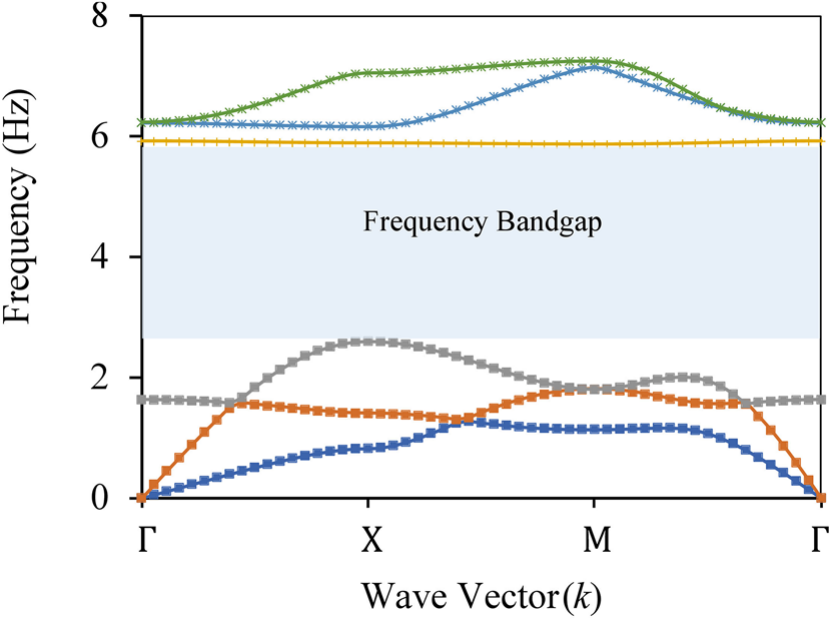
Dispersion relation of rubber plumbum unit cell.

For the 2 m unit cell made up of rubber and plumbum, at *p* = 0.55 lower bound frequency of the attenuation zone is 2.6 Hz and the upper bound frequency is 5.9 Hz, as shown in Fig. 8.

In this study, two different unit cells, rubber-steel with *p* of 0.75 and rubber-plumbum with *p* of 0.55, are used to make the periodic foundation. When both the unit cells are arranged in a multi-layered combination to make one finite composite periodic panel, it should yield a wide attenuation (band gap) zone from 2.6 Hz to 7.8 Hz.

## Attenuation zones of finite periodic panel

**Figure 9:**
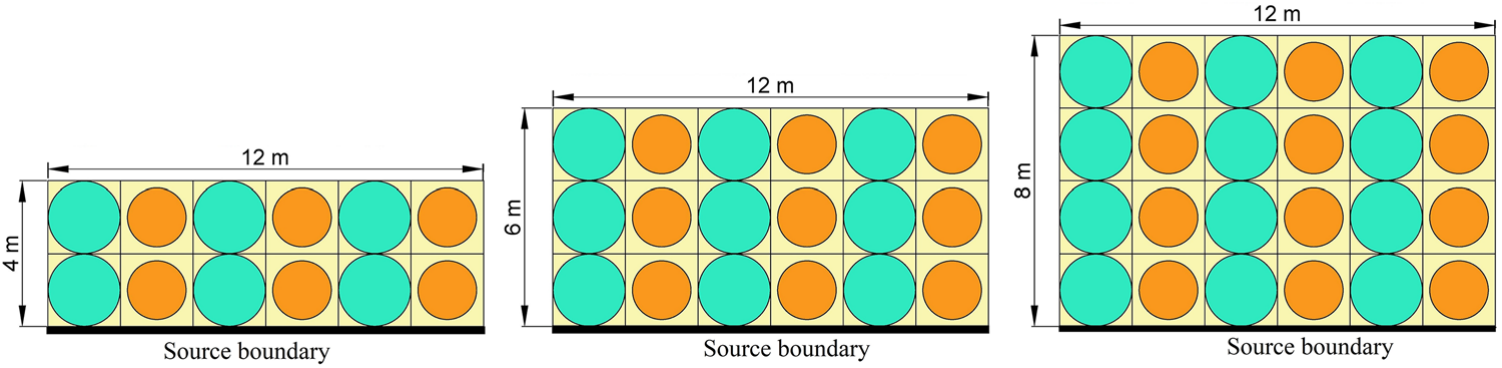
Periodic panels with a different number of vertical layers (bold base line represents the boundary of source excitation).

In this study, two different unit cells, rubber-steel with *p* of 0.75 and rubber-plumbum with *p* of 0.55, are used to make one finite periodic panel, as shown in Fig. 9. The yellow region represents the rubber matrix. The green core represents the steel scatters, and the orange core represents plumbum scatterers.

To verify that the periodic panel shown in the above figure has its attenuation zone between 2.6 Hz to 7.8 Hz, the frequency response function given by Eq. (8) is used.

8 $$ FRF = 20 \times \log_{10} \left( {\frac{{\delta_{o} }}{{\delta_{i} }}} \right) $$

In the above equation, $\delta_{i}$ is the input applied and $\delta_{o}$ is the output measured. When the FRF value is negative, it signifies output is less than input which indicates response reduction. The material properties are as per Table 1. A frequency domain study is used to provide harmonic displacement of unit amplitude at the base of the periodic panel swept over a range of frequencies from 0 to 30 Hz with a step size of 0.2 Hz. COMSOL Multiphysics version 5.2 has been used for simulation in the solid mechanics domain. For FRF computation, unit harmonic excitation is applied at the base of the periodic panel shown in Fig. 9 in the *x*-direction by fixing movement in the *y*-direction, and output is measured at the middle point of the top edge of the periodic panel. Low reflecting boundary conditions are applied at the left and right edges of the periodic panel. Gravitational forces are also considered on the whole metamaterial foundation. Equation of low reflecting boundary condition and gravitational force is represented by Eqs. (9) and (10), respectively.

9 $$ \sigma \cdot N = - \rho c_{p} \left( {\frac{\partial u}{{\partial t}} \cdot N} \right)N - \rho c_{s} \left( {\frac{\partial u}{{\partial t}} \cdot T} \right)T $$

10 $$ a = - g $$

where *N* and *T* are normal and tangential vector, respectively. *σ* is a force vector. *c*_*p*_ and *c*_*s*_ are the speed of pressure and shear waves in the material, respectively. *ρ* is density of material and *u* is the velocity vector.

**Figure 10:**
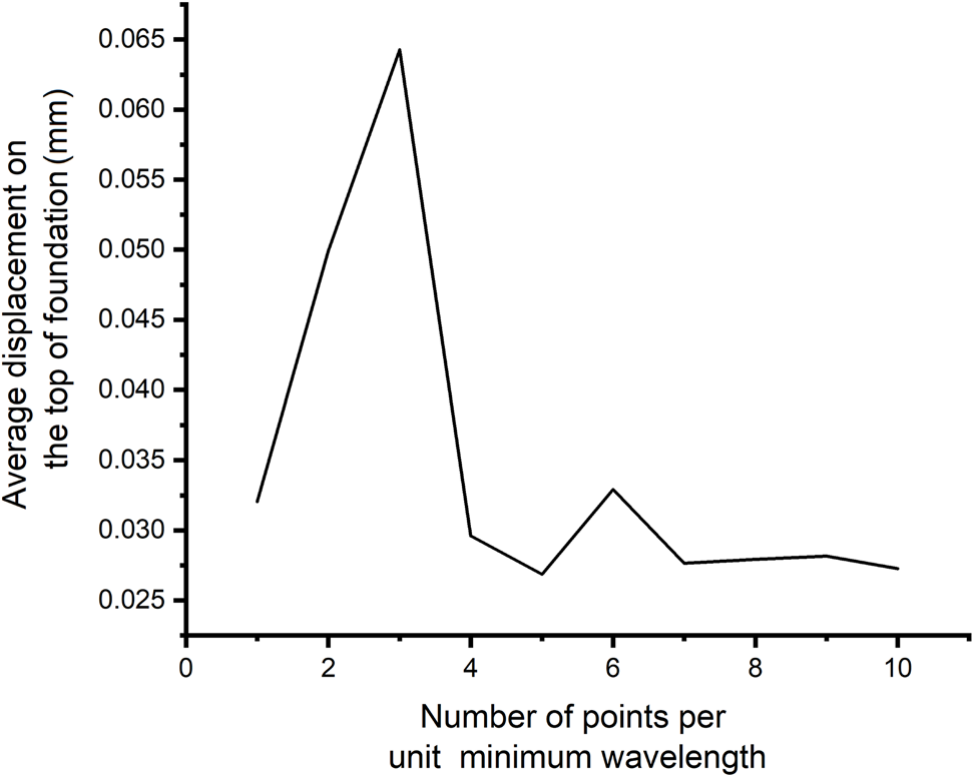
Convergence study.

**Figure 11:**
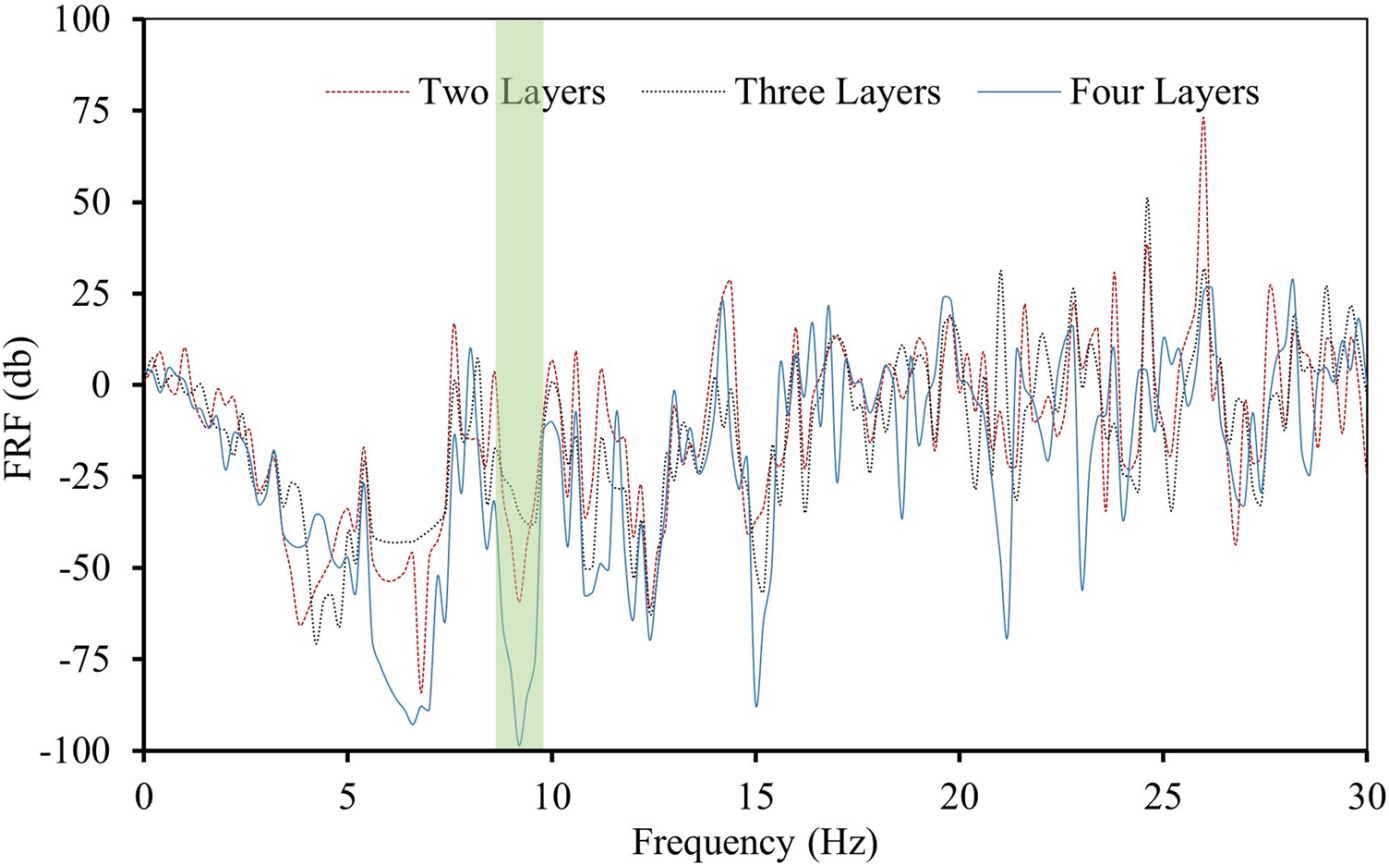
FRF graph of the periodic panel with a finite number of unit cells.

The FE analysis has been checked for convergence study as well, which is represented by Fig. 10. The solution converges at eight points per unit minimum wavelength. So, eight points per unit minimum wavelength is considered as a maximum size of tetrahedral element in the finite element simulation. FRF vs frequency graph obtained from the analysis is shown in Fig. 11.

Figure 11 shows a dip in the value of FRF between the frequencies ranging from 2.6 Hz to 7.8 Hz, which means the periodic panel used in the study can attenuate waves whose frequencies lie in the bandgap region of both the unit cells used to make the periodic panel. The Orange shaded region in Fig. 11 shows the attenuation zone of rubber-plumbum unit cell whereas the green shaded area shows the attenuation zones of the rubber-steel unit cell.

## Transient analyses

To check the effectiveness of the metamaterial foundation, its response is analysed under pre-recorded earthquake data as given in Table 2. Ground acceleration records of the earthquakes are taken from Pacific Earthquake Engineering Research Centre (PEER) ground motion database^24^.

**Table 2:** Past recorded earthquakes used in the study.

Earthquake (year of occurrence)	Record station	Magnitude	Peak ground acceleration (g)
Imperial Valley-02 (1940)	El Centro Array #9	6.95	0.2107
Kobe (1995)	Kobe University	6.9	0.274
Kern County (1952)	LA Hollywood Stor FF	7.36	0.0422

**Figure 12:**
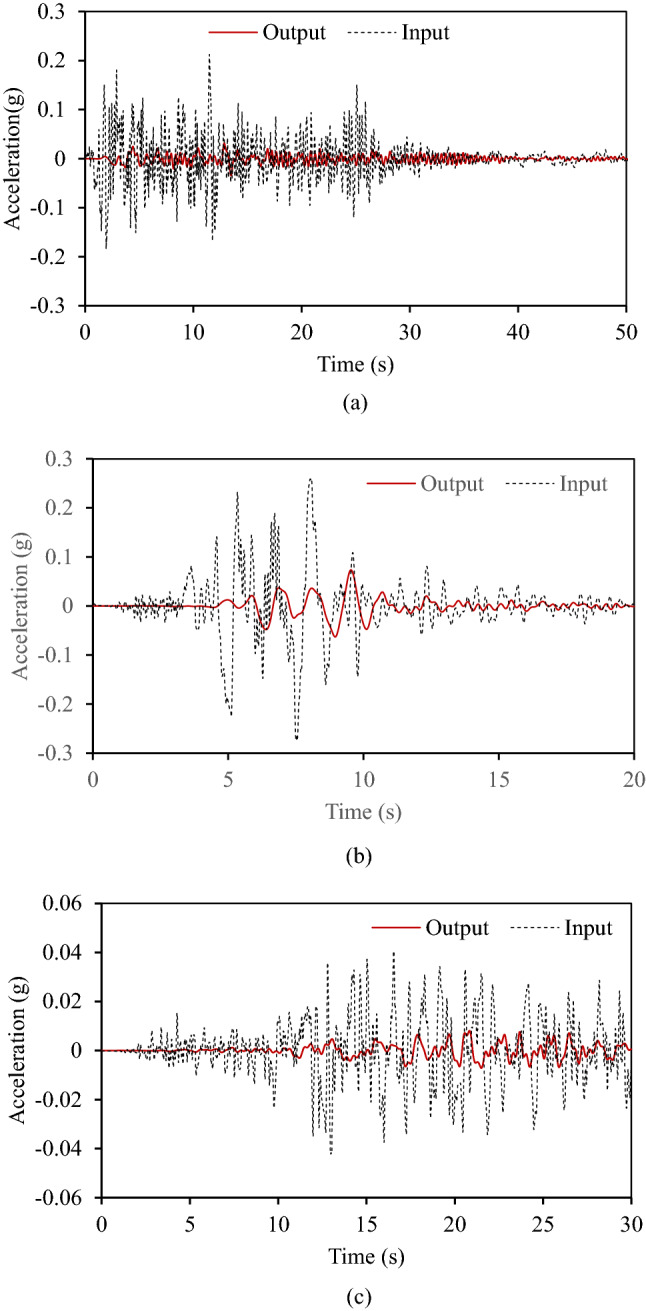
Horizontal acceleration response at the top (output) and bottom (input) of the metamaterial foundation in time domain under (**a**) Imperial Valley Earthquake, (**b**) Kobe earthquake, (**c**) Kern County Earthquake.

Seismic excitation is applied at the base of the periodic panel shown in Fig. 9 in the *x*-direction by fixing movement in the *y*-direction, and output is measured at the middle point of the top edge of the periodic panel. Low reflecting boundary conditions are applied at the left and right edges of the periodic panel. Response of metamaterial foundation under three different earthquake excitations is shown in Fig. 12.

**Figure 13:**
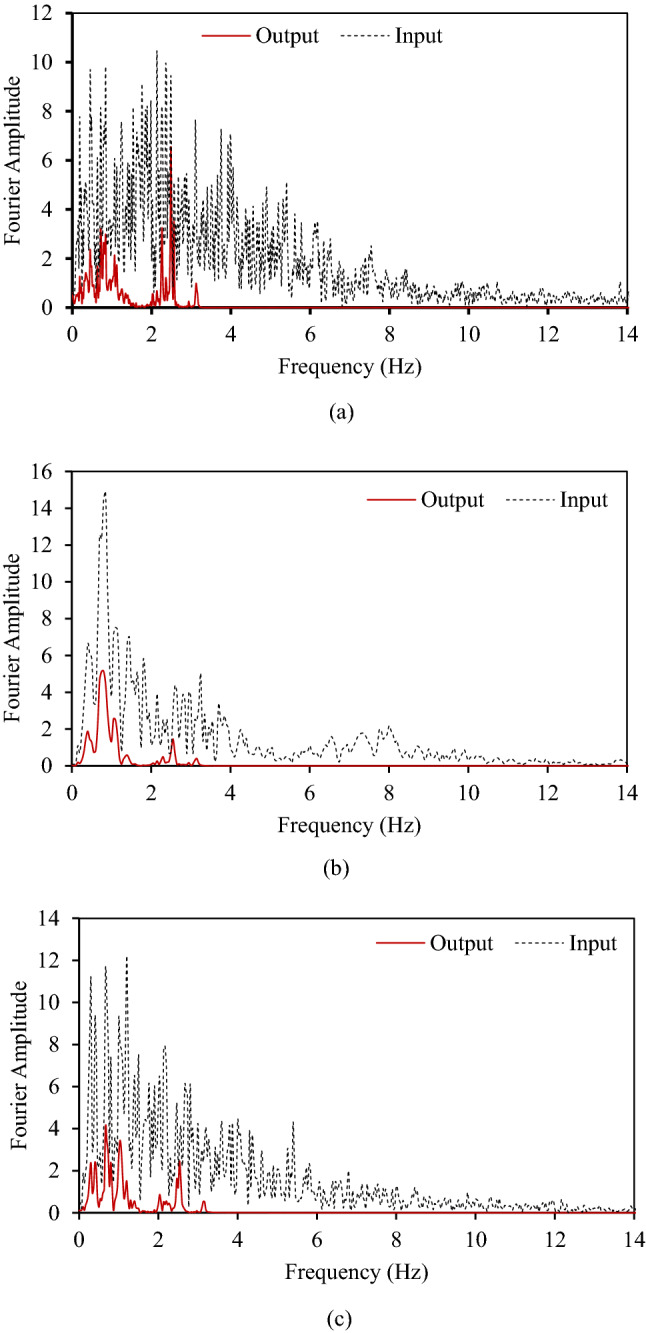
Horizontal acceleration response at the top (output) and bottom (input)of the metamaterial foundation in frequency domain under (**a**) Imperial Valley Earthquake, (**b**) Kobe earthquake, (**c**) Kern County Earthquake.

Figure 12 shows a significant reduction in the acceleration values measured at the top of the metamaterial foundation compared to acceleration values provided at the bottom. This means the metamaterial foundation can attenuate incoming seismic waves. To further analyse the response of metamaterial foundation, frequency domain analyses are done using Fast Fourier Transform. Figure 13 shows the response of the metamaterial foundation in the frequency domain.

**Figure 14:**
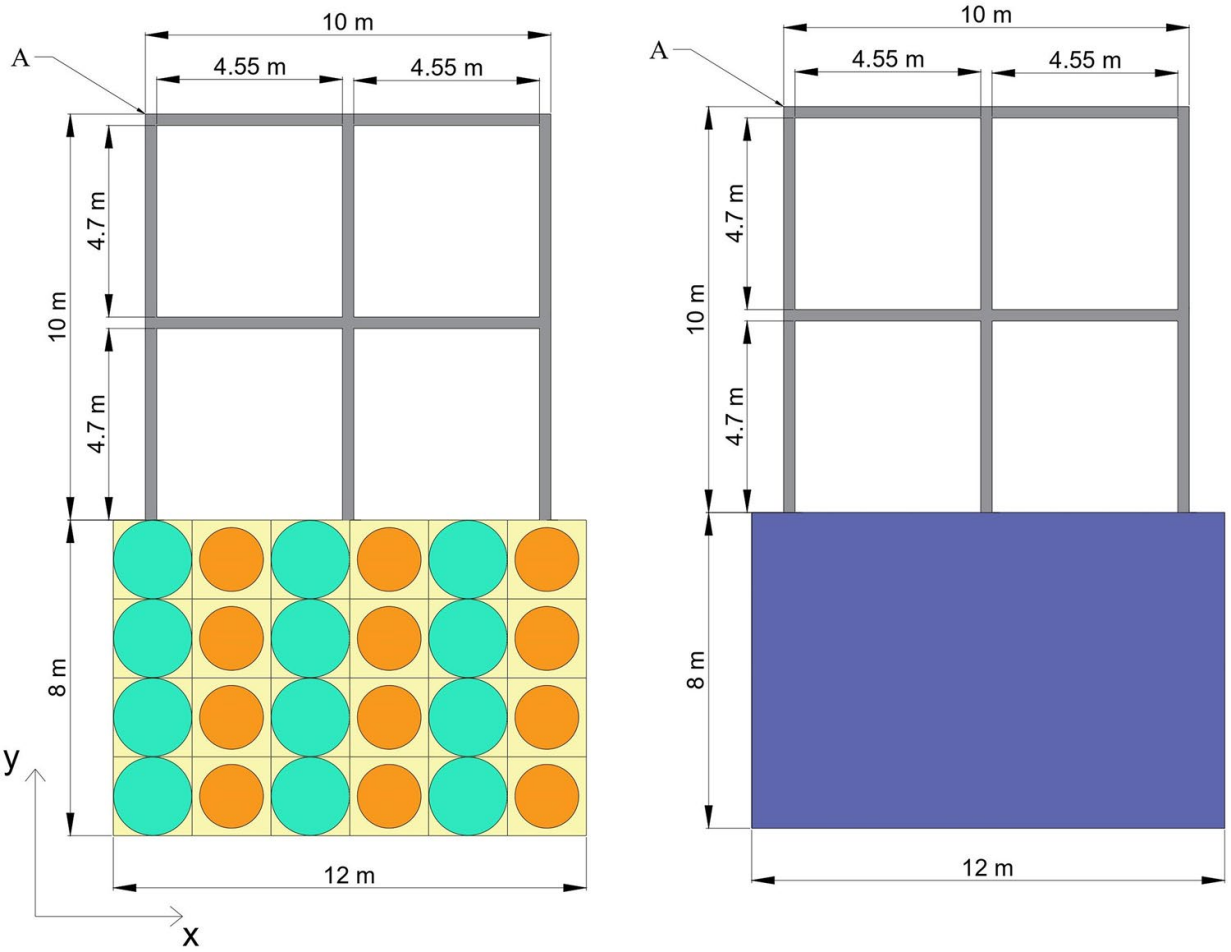
Steel frame on (**a**) metamaterial foundation, (**b**) concrete foundation.

To check how good metamaterial foundation as compared to concrete foundation is, a steel frame having natural frequency of 4.5 Hz is modelled on both metamaterial foundation and concrete foundation as shown in Fig. 14.

**Figure 15:**
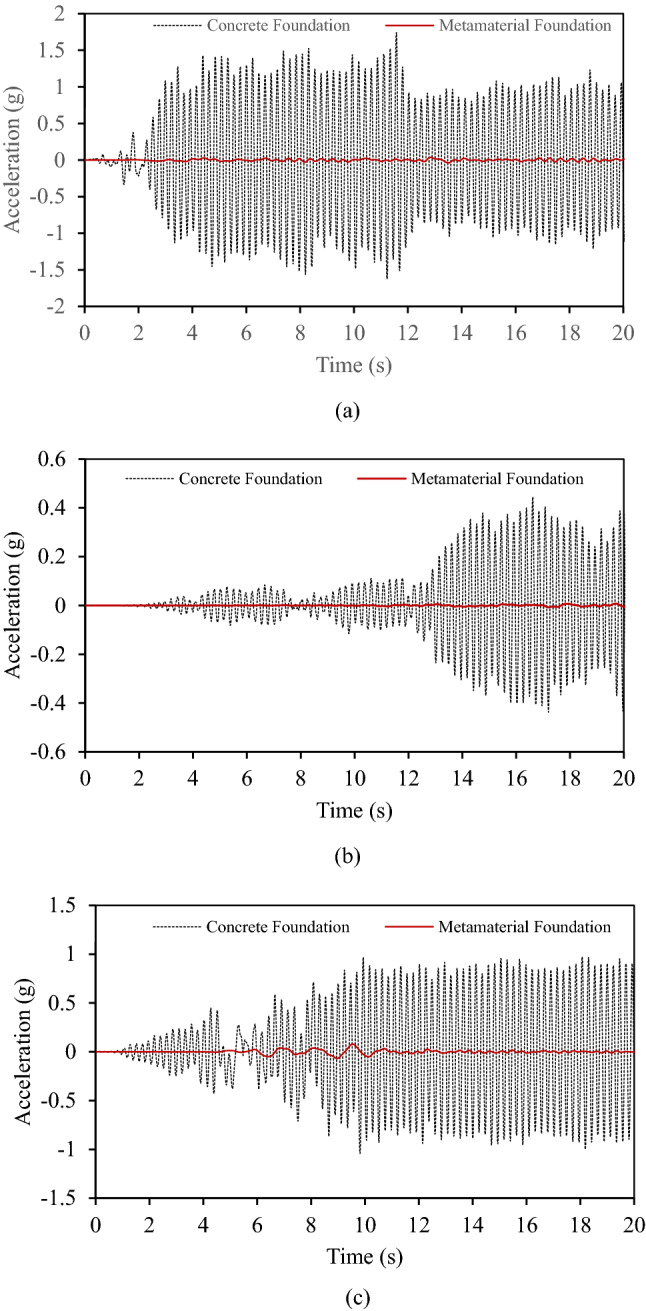
Horizontal acceleration response at point A of steel frame placed on metamaterial and concrete foundation one by one under (**a**) Imperial Valley Earthquake, (**b**) Kobe earthquake, (**c**) Kern County Earthquake.

Earthquake excitations are applied to the base of both foundations in the *x*-direction by fixing movement in the *y*-direction, and output is measured at point A on the steel frame. Low reflecting boundary conditions are applied on the left and right edges of the foundation. The responses of the frame to the applied excitations are measured at point A for both the foundations as shown in Fig. 15, are in the time domain.

One can see from Fig. 15 that in the case of the metamaterial foundation, the frame's response to exposed excitation is substantially reduced as compared to the frame on the concrete foundation in all three cases of earthquake excitations.

A comparative analysis of results from some of the related works and the present study is presented below in Table 3.

**Table 3:** Performance advantage table.

S. No.	Description of previous works	Advantages of current work
1	Cheng, Z., & Shi, Z.^22^ investigated bandgap zones of two-dimensional periodic structures consisting of two and three distinct materials promising the lowest attenuation zones in the range of 7.67 to 14 Hz	The presented work studies the effect of composite two-dimensional periodic structures, successfully producing low-frequency attenuation zones from 2.6 Hz to 7.8 Hz
2	Cheng, Z., & Shi, Z.^21^ studied and compared two-dimensional periodic panels for Bragg-scattering and Local-resonant periodic panels with different scatterers geometries such as square, rectangle, and circular A frequency domain (FRF) was studied to measure the effectiveness of periodic panels to mitigate waves/vibrations in the bandgap zone	The current work carried out the parametric study for two Bragg scattering periodic structures with circular scatter to identify a unique geometry leading to the lowest and widest frequency bandgaps possible The potential of periodic panels made up of these periodic structures to attenuate waves was carried out by using both the frequency-domain (FRF) and the time-domain (transient) analysis
3	Cheng, Z., & Shi, Z.^25^ proposed a novel multidimensional, local-resonant composite periodic foundation for seismic isolation The composite periodic foundation was fabricated by using two periodic structures (composed of three materials) in several vertical layers. The frequency attenuation zones obtained were approximately (4.08 Hz–4.94 Hz) and (7.94 Hz–15.1 Hz). They conducted harmonic and time-history analysis on the composite periodic structure/foundation and compared the attenuation effect with the traditional concrete foundation	This work identified the geometry of two distinct Bragg-scattering periodic structures (composed of two materials) yielding the bandgaps with low-frequency attenuation zones, which can effectively mitigate a real-time earthquake The obtained frequency bandgaps for both the periodic structures were (2.6 Hz–5.9 Hz) and (5.2 Hz–7.8 Hz). To widen the attenuation frequency range or combine the two bandgaps, the authors made a horizontal layer of the periodic structures by placing two different unit cells adjacently. The horizontal layers were then stacked vertically to form a novel periodic foundation Its efficiency in mitigating waves was measured using harmonic analysis (FRF) with different vertical layers. Further, time-history analyses were carried out with several pre-recorded earthquake data on the periodic structures The results show that the composite periodic foundation achieved satisfactory wave attenuation from 2.6 Hz to 7.8 Hz. The composite periodic foundation achieved a novel multi-range low-frequency attenuation zone

## Conclusion

The presented work aims to analyse the attenuation zones of the metamaterial foundation and find the possible ways to lower the frequency range of the attenuation zones to attenuate real-time earthquakes. This work has identified the geometry of two distinct Bragg-scattering periodic structures (composed of two materials) yielding the bandgaps with low-frequency attenuation zones, which can effectively mitigate a real-time earthquake. The obtained bandgaps for both the periodic structures form an overlapping range from 2.6 Hz to 5.9 Hz and 5.2 Hz to 7.8 Hz. To combine the band width of these two bandgaps, the authors made a horizontal layer of the periodic structures by placing two different unit cells adjacently. The horizontal layers are stacked vertically to form a novel periodic foundation. Therefore, a periodic structure with a wider attenuation zone can be achieved by using a combination of distinct unit cells structured in multiple layers. In the case where the excitation frequency lies within the attenuation zone of a periodic structure, simulation results show a significant reduction in wave amplitude. Its efficiency to mitigate the seismic waves is measured using harmonic analysis with different vertical layers. Further, the transient analysis is carried out with several pre-recorded earthquake data on the periodic structures.

To verify the accuracy of the computation, the band gap is validated with the existing literature. The results reveal that the attenuation zones of the periodic structure depend on the unit cell's geometry and its material properties. By increasing the unit cell size, band gaps in promising lower frequency regions can be achieved. The effect of materials and the geometric properties of the unit cell on attenuation zones are also discussed. Simulation results show that periodic structures could be designed as the foundation of structures to attenuate seismic waves. It is found that metamaterial foundations subjected to different ground acceleration excitations effectively attenuate ground accelerations. A comparative study between concrete and metamaterial foundations reveals that metamaterial foundations can significantly reduce the response of the steel frame as compared to the concrete foundation. The results show that the composite periodic foundation achieved satisfactory wave attenuation from 2.6 Hz to 7.8 Hz. This low frequency and wide band gap is a significant contribution which can help design future metamaterial foundation to mitigate the effect of earthquake excitation.

## Supplementary Information

Supplementary Information.

## Data Availability

The datasets generated and/or analysed during the current study are not publicly available due to ongoing research but are available from the corresponding author on reasonable request.
